# Utilization of a Web-Based vs Integrated Phone/Web Cessation Program Among 140,000 Tobacco Users: An Evaluation Across 10 Free State Quitlines

**DOI:** 10.2196/jmir.3658

**Published:** 2015-02-04

**Authors:** Chelsea M Nash, Katrina A Vickerman, Elizabeth S Kellogg, Susan M Zbikowski

**Affiliations:** ^1^Alere WellbeingResearch, Training and Evaluation ServicesSeattle, WAUnited States

**Keywords:** Internet-based intervention, tobacco cessation, smoking cessation, Internet, telephone, behavior, evaluation studies, online support

## Abstract

**Background:**

Phone-based tobacco cessation program effectiveness has been established and randomized controlled trials have provided some support for Web-based services. Relatively little is known about who selects different treatment modalities and how they engage with treatments in a real-world setting.

**Objective:**

This paper describes the characteristics, Web utilization patterns, and return rates of tobacco users who self-selected into a Web-based (Web-Only) versus integrated phone/Web (Phone/Web) cessation program.

**Methods:**

We examined the demographics, baseline tobacco use, Web utilization patterns, and return rates of 141,429 adult tobacco users who self-selected into a Web-Only or integrated Phone/Web cessation program through 1 of 10 state quitlines from August 2012 through July 2013. For each state, registrants were only included from the timeframe in which both programs were offered to all enrollees. Utilization data were limited to site interactions occurring within 6 months after registration.

**Results:**

Most participants selected the Phone/Web program (113,019/141,429, 79.91%). After enrollment in Web services, Web-Only were more likely to log in compared to Phone/Web (21,832/28,410, 76.85% vs 23,920/56,892, 42.04%; *P*<.001), but less likely to return after their initial log-in (8766/21,832, 40.15% vs 13,966/23,920, 58.39%; *P*<.001). In bivariate and multivariable analyses, those who chose Web-Only were younger, healthier, more highly educated, more likely to be uninsured or commercially insured, more likely to be white non-Hispanic and less likely to be black non-Hispanic, less likely to be highly nicotine-addicted, and more likely to have started their program enrollment online (all *P*<.001). Among both program populations, participants were more likely to return to Web services if they were women, older, more highly educated, or were sent nicotine replacement therapy (NRT) through their quitline (all *P*<.001). Phone/Web were also more likely to return if they had completed a coaching call, identified as white non-Hispanic or “other” race, or were commercially insured (all *P*<.001). Web-Only were less likely to return if they started their enrollment online versus via phone. The interactive Tobacco Tracker, Cost Savings Calculator, and Quitting Plan were the most widely used features overall. Web-Only were more likely than Phone/Web to use most key features (all *P*<.001), most notably the 5 Quitting Plan behaviors. Among quitlines that offered NRT to both Phone/Web and Web-Only, Web-Only were less likely to have received quitline NRT.

**Conclusions:**

This paper adds to our understanding of who selects different cessation treatment modalities and how they engage with the program in a real-world setting. Web-Only were younger, healthier smokers of higher socioeconomic status who interacted more intensely with services in a single session, but were less likely to re-engage or access NRT benefits. Further research should examine the efficacy of different engagement techniques and services with different subpopulations of tobacco users.

## Introduction

Fifty years after the release of the first Surgeon General’s Report on Smoking and Health, tobacco use is still the leading preventable cause of death in the United States [1]. Although cigarette use in particular has declined among American adults in the past 20 years, this shift is driven by a small proportion of relatively higher-income counties [2], indicating a widespread need for accessible and affordable cessation services. Over the past 2 decades, state governments throughout the United States have provided phone-based tobacco cessation services, called quitlines, to help tobacco users quit by providing evidence-based counseling. These services are offered statewide at no charge and often include nicotine replacement therapy (NRT). More recently, quitlines have responded to the aforementioned need for more easily accessible services by offering Web programs for use not only alongside traditional phone-based programs, but also as a stand-alone service without phone-based counseling. According to North American Quitline Consortium (NAQC) data [3], 44 state quitlines offered a self-directed Web-based intervention in 2012 [4], representing a notable increase from 27 in 2010 [5]. NAQC does not specify whether these Web services are stand-alone or integrated with standard quitline phone services, highlighting the need for more research into the structure of these services.

Tobacco users want to access cessation help via the Internet for a number of reasons, including convenience and a desire to remain anonymous [6]. Users also have reported a desire to access personalized, interactive websites, which are less common than sites simply containing educational content related to tobacco cessation [7]. State health departments have an interest in implementing Web-based services because they have been associated with the lowest cost per quit when compared to treatment delivered via phone or in person at a health care clinic or workplace [7]. Web-based services also have the potential to combine the wide reach of Internet-based health promotion with aspects of face-to-face counseling; those Web-based interventions that mirror other counseling modalities with a tailored, interactive approach have been shown to be more effective [8,9].

Despite the proliferation of Web-based services and the study of reasons for use, relatively little is known about who uses Web-only services, especially when users are given the choice between different programs. Randomized controlled trials have provided support for the efficacy of some Web-based cessation services [10], but more research is needed to understand how tobacco users select a treatment modality, their demographics, and how they engage with treatments in a real-world setting of tobacco users seeking help with quitting. The purpose of this paper is to examine the characteristics, utilization patterns, and return rates of tobacco users self-selecting into 1 of 2 free state tobacco cessation programs: stand-alone Web services versus Web services offered in combination with phone-based counseling. This information could inform outreach strategies, content tailoring, and future research evaluating outcomes for different program types.

## Methods

### Study Design

In this real-world observational study, participants selected 1 of 2 tobacco cessation programs offered through their state quitlines: (1) an integrated phone/Web program (Phone/Web) or (2) a stand-alone Web program (Web-Only). The Western Institutional Review Board reviewed the study and determined that it met the requirements for a waiver of consent under 45 CFR 46.116(d) on March 20, 2014.

### Participants and Sample Selection

Ten state quitlines that offered both (1) a phone-based 1-call or multiple-call cessation program integrated with the Web Coach website (Phone/Web) and (2) a stand-alone Web Coach website program (without coaching calls; Web-Only) for the majority of the study timeframe were invited and agreed to participate in the study: Connecticut, Delaware, Florida, Idaho, Indiana, Kansas, New Mexico, North Carolina, Oklahoma, and Oregon. English-speaking participants aged 18 years and older who enrolled in a Phone/Web or Web-Only program through 1 of the 10 state quitlines from August 2012 through July 2013 were included in this analysis; for each state, registrants were only included from the timeframe in which both programs were offered to all enrollees.

Based on these inclusion criteria, 149,362 registration records were identified, of which 6698 individuals (4.48%) had 2 or more program enrollments during the study timeframe. To represent individuals only once in the analysis groups, the following steps were taken to remove duplicate registrations:

1. For participants with multiple Phone/Web program enrollments (4620/149,362, 3.09%), the first enrollment was retained to include the less biased case.
2. Because each individual who enrolled in Web-Only was intended to have only 1 Web Coach website account for life, those participants with multiple Web-Only program enrollments (623/149,362, 0.42%) were duplicated in error. In light of this, the enrollment with the greatest number of log-in days was retained to include the most accurate and complete data.
3. For participants who enrolled in the Web-Only program and switched to a Phone/Web program soon after (1394/149,362, 0.93%), the phone program enrollment was retained.
4. The most appropriate record could not be determined for the remaining participants (380/149,362, 0.25%) who had enrolled in both the Phone/Web and Web-Only programs. Because this group amounted to a very small percentage of the final sample, these participants were excluded from all analyses.

Analyses focused on the 141,429 unique adult English-speaking participants who enrolled in an integrated Phone/Web (113,019/141,429, 79.91%) or Web-Only (28,410/141,429, 20.09%) program offered by 1 of the 10 participating state quitlines. All participating states had contracted with Alere Wellbeing for the services offered through the quitline (including phone counseling, mailed materials, and Web-based services).

### Phone/Web and Web-Only Program Descriptions

#### Enrollment

Quitline participants started their enrollment (“method of entry”) in the cessation program online, over the phone by calling the quitline, or via fax referral, a process in which health care providers fax-referred their patients who were then proactively called by the quitline. Participants who started enrollment online could also request a callback to complete their enrollment with a registration specialist by phone. During both phone and Web enrollment procedures, participants were presented with the program options available to them and then selected their preferred program.

Participants in both the Phone/Web and Web-Only programs enrolled in the Web Coach website by providing their email address and consenting to be contacted via email. Participants then had to authenticate their account by using the log-in information provided in the initial email sent to them by the program.

#### Phone Program

The phone-based coaching program (Phone/Web) operated by Alere Wellbeing was offered as a 1-call or multiple-call program. The 1-call program included an initial assessment and planning call with Quit Coach staff to identify the participant’s strengths and challenges, and to develop a quit plan. The multiple-call program included all aspects of the 1-call program plus either 3 or 4 outbound calls from the quitline. Participants in both phone programs were encouraged to call their quitline for support as needed. Both phone programs also included written educational materials for the participant (Quit Guide), referrals to community resources (when requested), health plan information (when appropriate), and access to the Web Coach website.

#### Web Coach Website

The Web Coach 2.0 website is the second version of the online participant application for the tobacco cessation coaching program operated by Alere Wellbeing (1.0 launched in 2006; 2.0 launched July 2011). It is grounded in social cognitive theory and designed to guide tobacco users through an evidence-based process of quitting tobacco. The website was offered as a stand-alone program (Web-Only) or integrated with the phone-based coaching program described previously (Phone/Web) and was tailored to each participant’s tobacco status and needs (ie, different content was recommended and enabled based on the participant’s quit status and activities completed). The Web Coach website also allowed participants to reach out to Quit Coach staff through phone call requests (for those in the Phone/Web program) and through chat and email in both Phone/Web and Web-Only; Quit Coach staff also moderated and participated in community forum discussions on the site. However, the focus of this paper is on the utilization of Web-based features and not the counseling options with a coach.

**Figure 1:**
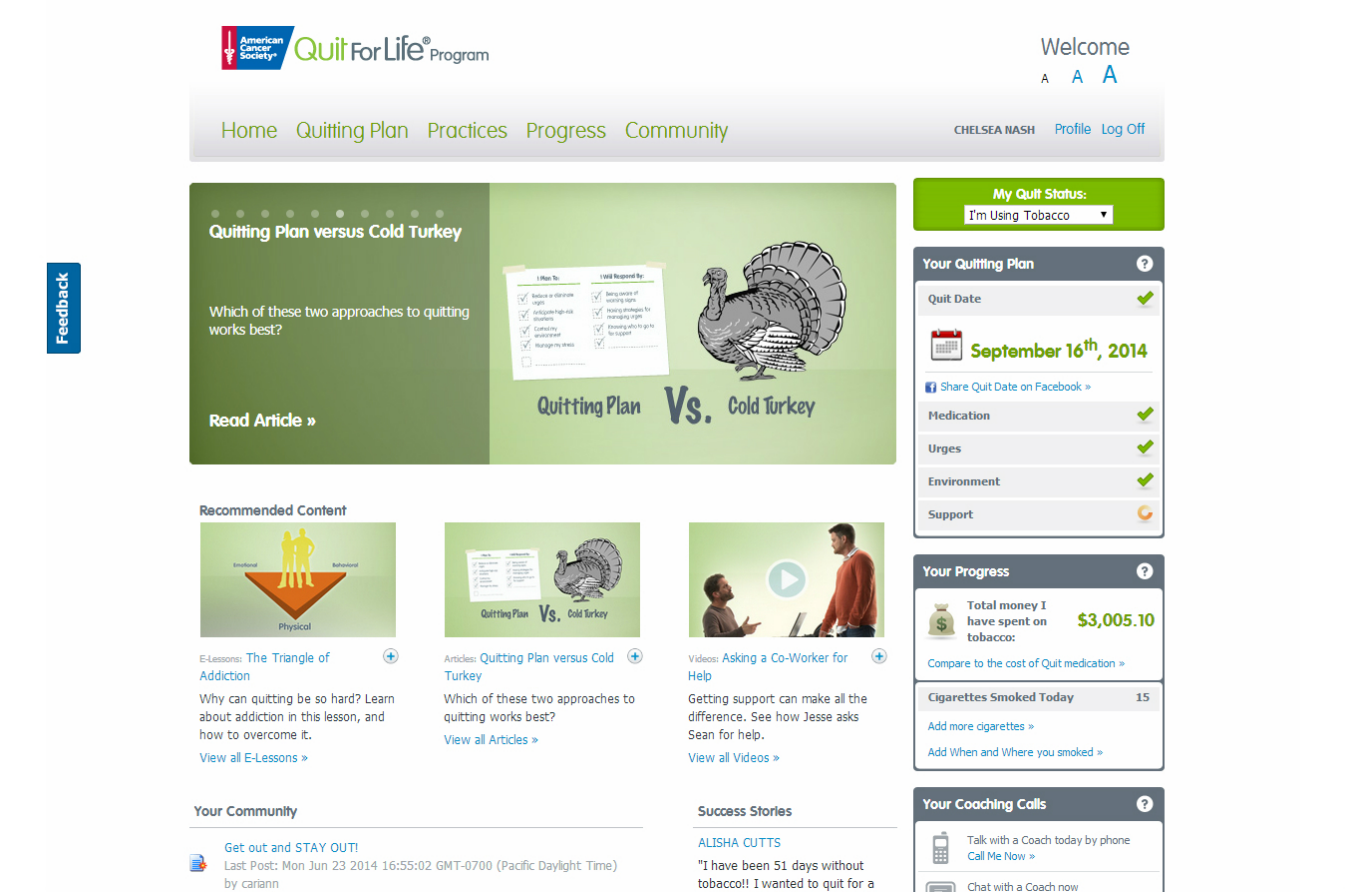
Screenshot of Web Coach website home page.

**Figure 2:**
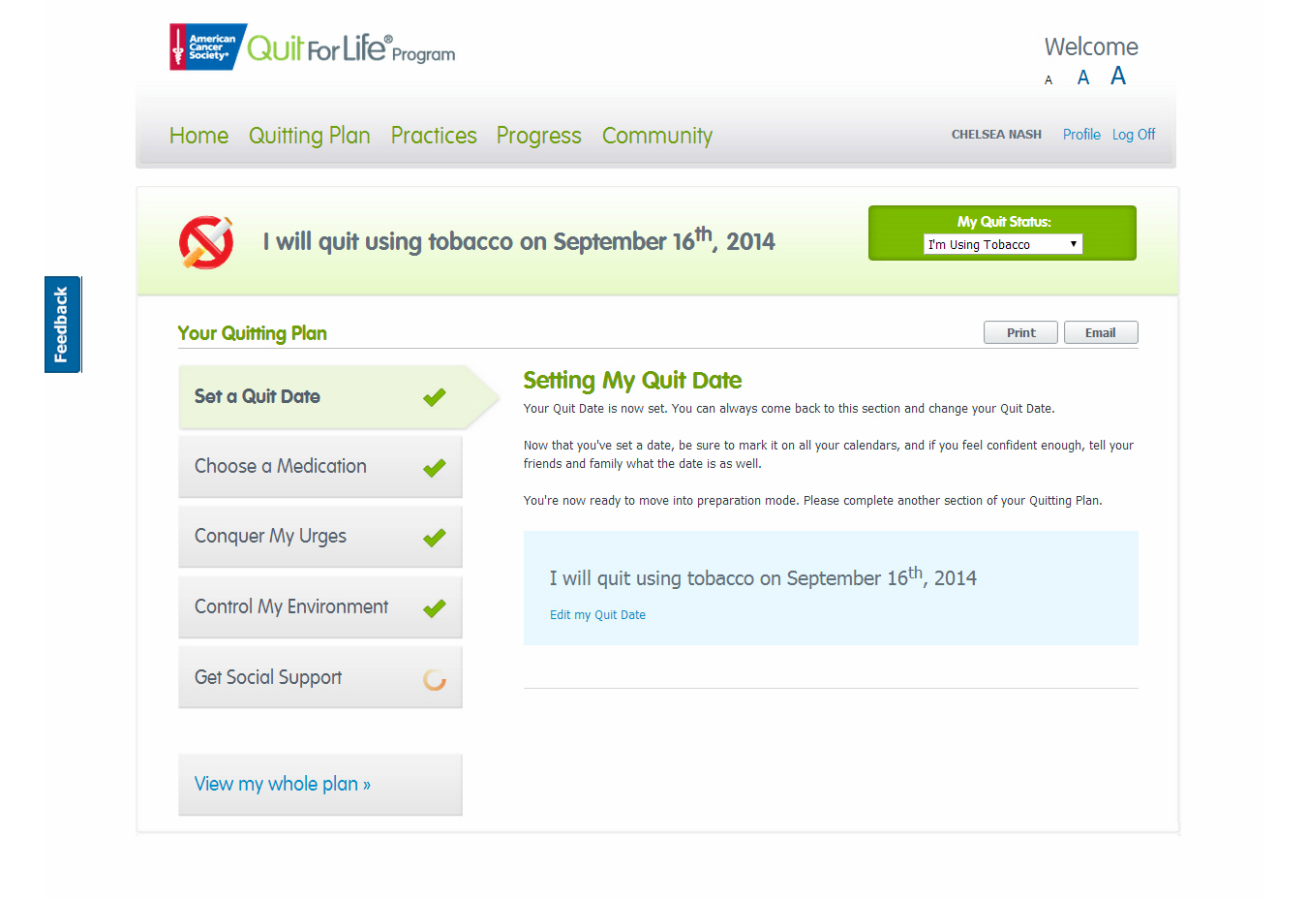
Screenshot of Quitting Plan webpage: setting a quit date.

**Figure 3:**
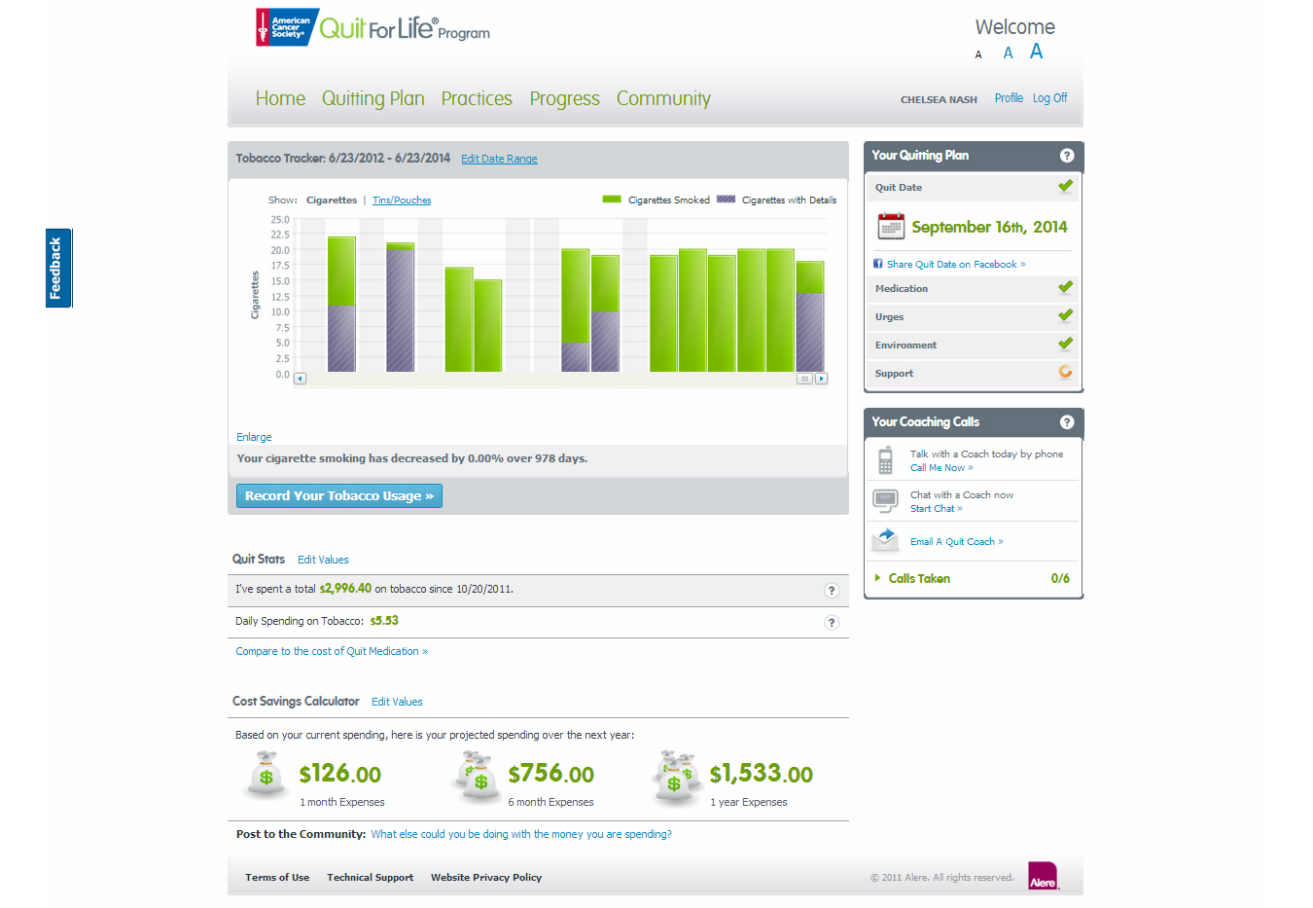
Screenshot of Progress webpage with Tobacco Tracker and Cost Savings Calculator for participants who reported that they were still using tobacco.

**Figure 4:**
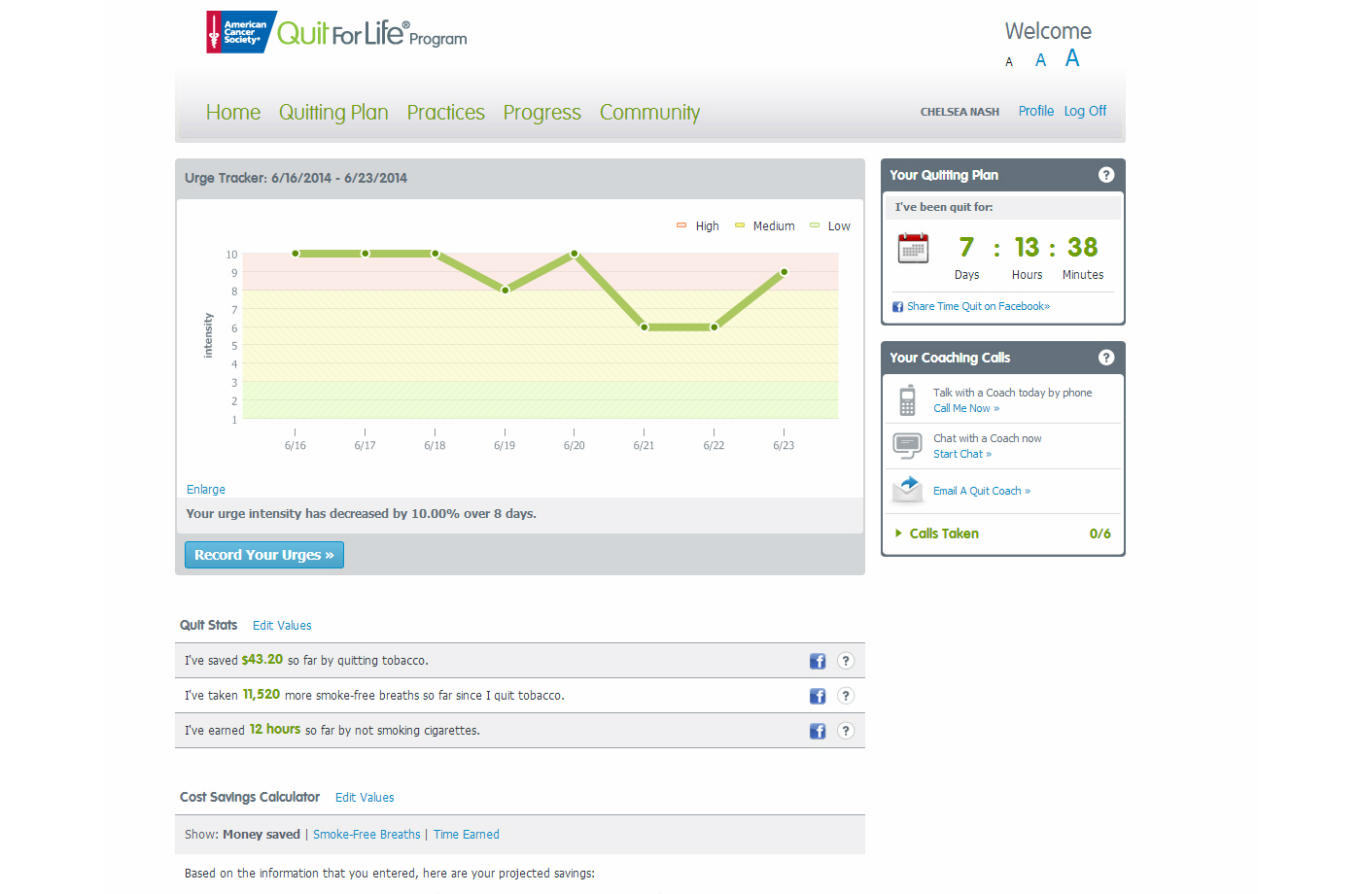
Screenshot of Progress webpage with Urge Tracker for participants who reported that they had quit using tobacco.

**Figure 5:**
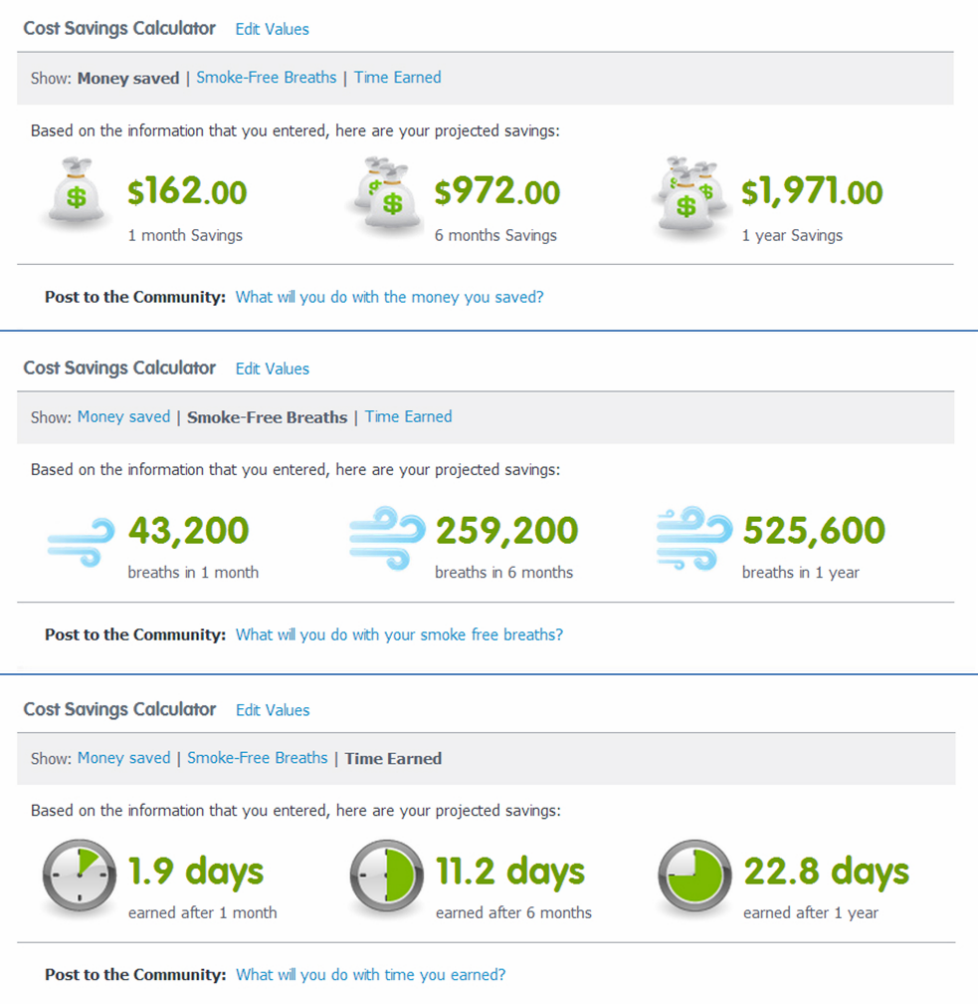
Screenshot of Progress webpage with Cost Savings Calculator for participants who reported that they had quit using tobacco. Participants could select money saved, smoke-free breaths, or time earned.

**Figure 6:**
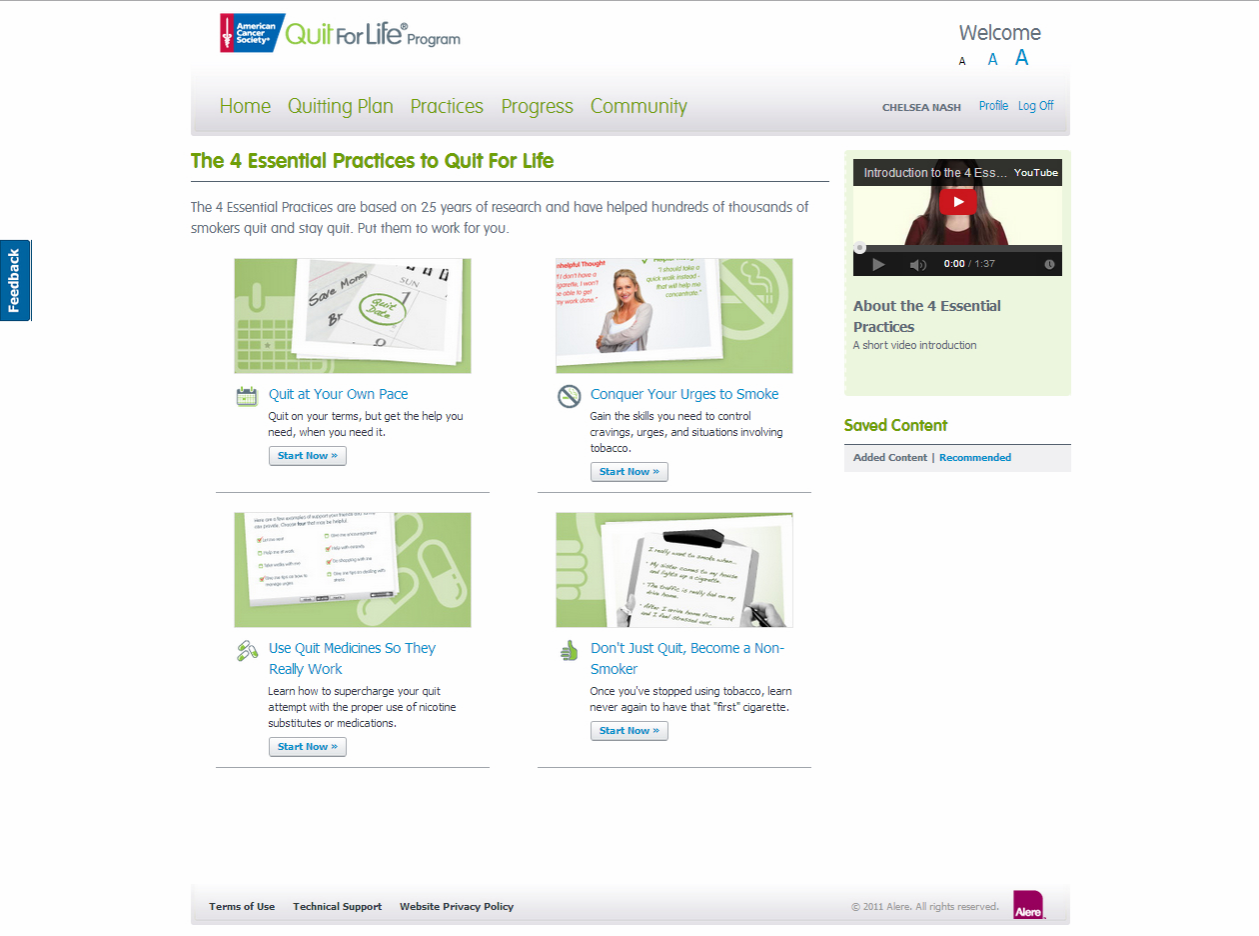
Screenshot of Practices webpage.

**Figure 7:**
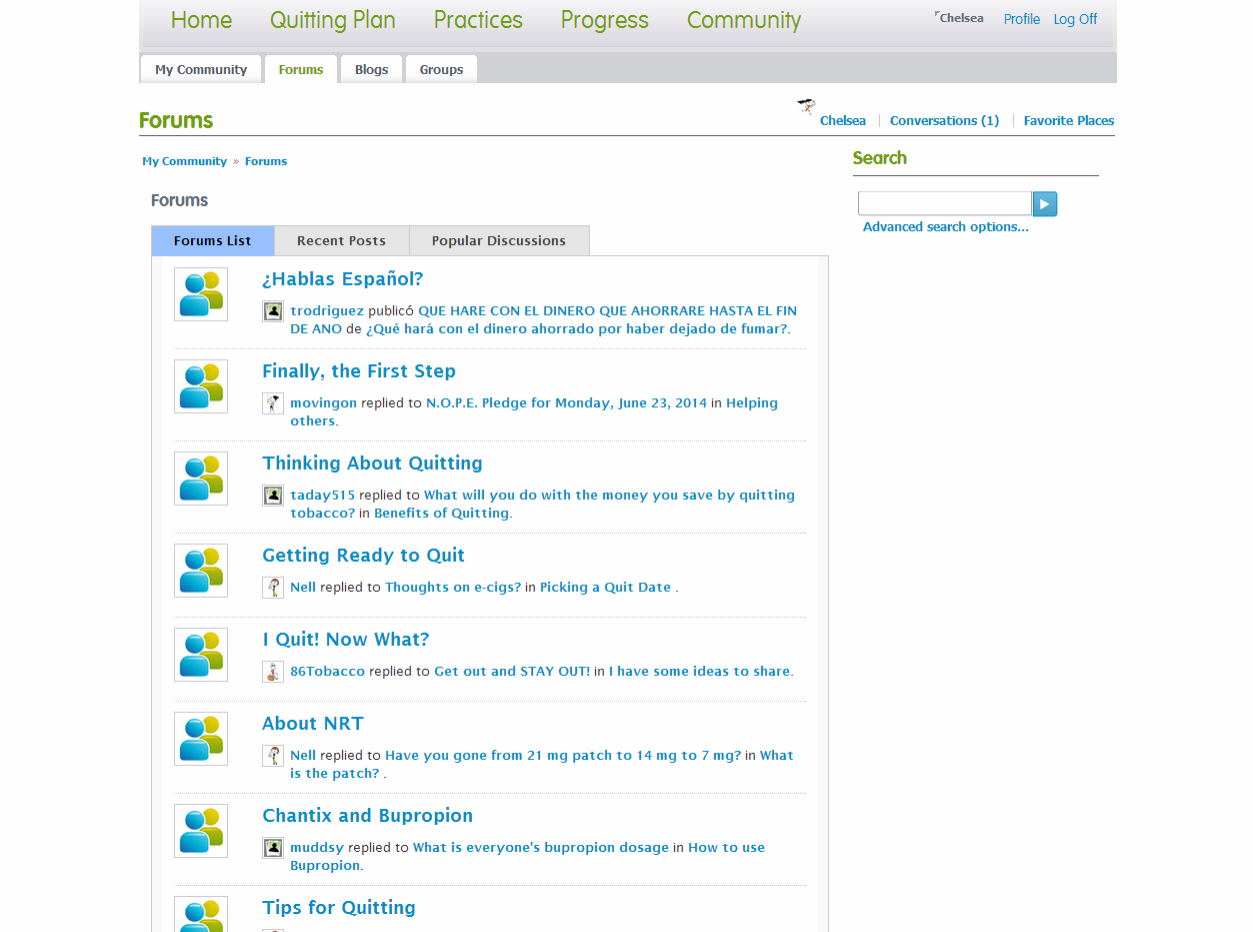
Screenshot of Community area.

The Web Coach website home page (Figure 1) included links to recommended site content for each participant. The 4 key groups of features included:

1. The Quitting Plan (Figure 2): an interactive tool that enabled participants to build a personalized plan to quit using tobacco. The Quitting Plan guided participants through choosing a quit medication, setting a quit date, conquering urges and cravings to use tobacco, controlling their environment, and getting social support.
2. Progress Trackers: tools that helped participants who had not quit record and track their smoking patterns (Tobacco Tracker) and the potential financial savings of quitting (Cost Savings Calculator) (Figure 3). Participants who quit could use the Urge Tracker to record the strength of their urges or cravings to use tobacco (Figure 4), and could use the Cost Savings Calculator to review time quit, money saved, smoke-free breaths taken, and free time gained by quitting (Figure 5).
3. Interactive Practice Content (Figure 6): the Practices page introduced the 4 Essential Practices of Quitting, where participants could access e-lessons, articles, videos, and worksheets based on the Practices.
4. Community (Figure 7): the Community area was a place for participants to connect with one another to discuss their successes and challenges with quitting and staying quit; Quit Coach staff moderated the forums and actively participated in the discussions.

Web Coach website participants in both programs were sent the same tailored emails (up to approximately 25 messages) to remind and encourage them to log in to the site. First-time participants were sent a reminder email to visit if they had not logged in to the site within several days of enrolling in the program; additional reminder emails were sent if the participant still had not logged in at later time points. Participants who logged in but did not return within a certain time period received a reminder email to visit the site. Participants also received reminders to set a quit date, complete their quit plan, or update their tobacco status after their quit date if they had not done so. When a participant ordered NRT through the Web Coach website, summary information and links to use instructions were emailed. Emails also provided motivation through encouraging messages around the quit date, and through congratulatory emails when the participant reduced their tobacco use. The email schedule was designed to anticipate typical withdrawal symptoms and send encouragement to track urges and work on coping skills after the participant quit. Finally, regular check-in emails were sent to encourage participants to stay quit and follow their stay-quit plan.

#### State Offerings

All 10 states included in the study offered both a Web-Only program and an integrated Phone/Web program to all participants. Eight of 10 states offered the multiple-call program described previously to all adults (dependent on readiness to quit for some states). In 2 state quitlines, the multiple-call program was only available to select groups of registrants (eg, uninsured, Medicaid); other registrants in these quitlines were eligible for the 1-call program. Eight states also offered a 10-call program to pregnant tobacco users. During the study timeframe, 6 states offered NRT through both their Phone/Web and Web-Only programs, 2 states offered NRT to Phone/Web enrollees only, and 2 states did not offer NRT through either program.

### Measures

Demographic characteristics (gender, age, race/ethnicity, education, health insurance, chronic condition status) and tobacco use (type, frequency, amount, years of tobacco use, time to first use after waking, other users at home and/or work) data were collected during standard program registration that is compliant with the NAQC Minimal Data Set [11]. Participants who enrolled online were asked the same questions in a participant-facing Web enrollment process. Several state quitlines also collected custom demographic data during registration (marital status, income, sexual orientation, mental health condition status). Data that were not collected for all states are identified in the table notes.

Every unique participant who enrolls in the Web Coach website is intended to have access to the same account for life, regardless of the number of times the participant re-enrolls in a quitline program. To avoid a time-lapse bias, website utilization data for this study were limited to site interactions occurring in the 6 months (up to 185 days) after registration.

Web Coach website activity (interactions and feature use) was recorded and linked to unique participants automatically through Google Analytics and the website. Efforts were also made to record log-ins and the duration of each log-in session; however, Google Analytics was often blocked by individual users or employer networks, resulting in missing data in the log-in and session minutes fields. To circumvent this issue, engagement was determined by creating a log-in days variable, which counted 1 log-in day for every distinct date on which the participant completed a site interaction.

To classify study participants by engagement level, *users* were defined as participants who logged in to the Web Coach website at any time in the 6 months following registration. *Return users* were defined as anyone who logged in at any time in the 6 months following registration and then returned at a later date during the same 6-month time period (ie, logged in to the Web Coach website on at least 2 different days in the 6 months following registration).

### Statistical Analysis

Chi-square and *t* test analyses were used to examine differences in characteristics and Web utilization between Phone/Web and Web-Only enrollees, as well as differences in return rates between subpopulations within each program. Bivariate post hoc analyses were completed as necessary. Multivariable logistic regression analyses were used to examine predictors of program choice and return rates within each program. Models included predictor variables that were collected by all 10 states, had limited amounts of missing data, and measured participant characteristics (age, gender, race/ethnicity, education, insurance status, chronic condition status), tobacco dependence (time to first use, cigarettes per day), and method of program entry. A secondary model for program choice also included mental health condition status, which was only asked by 6 states; this secondary model was examined because mental health condition status was a significant predictor of program choice in bivariate analyses and data were available from the majority of states. Both return user models (within Phone/Web and Web-Only) also included receipt of quitline NRT as a predictor variable; analyses examining return users within Phone/Web additionally included phone treatment intensity (1-call vs multiple-call program) and call completion (0 calls vs ≥1 calls) as predictors. Because these behaviors (eg, call completion, NRT selection, selection of the multiple-call program) occurred after tobacco users selected the Phone/Web or Web-Only program in some or all cases, these variables were not included in the program choice model. State quitline was included as a fixed effect in every model to account for pre-existing differences in services and tobacco control policies within each state. All analyses were conducted in SAS v9.3 (SAS Institute, Inc, Cary, NC, USA).

Because of the large sample size, a number of findings were statistically, but not meaningfully, significant. We used a Bonferroni adjustment to account for the large number of statistical comparisons. Results are reported as significant where *P*<.0001 and the absolute difference in percentage points between comparison groups rounded to 5 or greater; this significance threshold was determined post hoc after initial review of analysis findings.

## Results

### Participant Characteristics

Overall, the majority of registrants were female (59.29%), mean age 44.4 (SD 13.8) years, white non-Hispanic (72.90%), heterosexual (93.86%), had a high school degree (27.40%) or higher (47.27%), but an annual household income of less than US$15,000 (51.45%), were uninsured (42.62%) or commercially insured (25.47%), and were daily cigarette smokers (94.02%) at a mean rate of 19 cigarettes per day (SD 11.3) (Table 1). Nearly one-half (48.32%) used tobacco within 5 minutes of waking at the time of enrollment, indicating high nicotine dependence. Approximately one-third (32.62%) reported at least 1 of 4 chronic health conditions (asthma, chronic obstructive pulmonary disease, coronary artery disease, and/or diabetes) and nearly one-half (46.59%) reported a mental health condition diagnosis.

**Table 1:** Characteristics of total sample and the Phone/Web and Web-Only programs (N=141,429^a^).

Baseline characteristic or program component			Total N=141,429	Phone/Web n=113,019	Web-Only n=28,410	*P*
**Gender, % (n/N)**						<.001
	Female		59.29 (83,833/141,386)	59.03 (66,695/112,981)	60.33 (17,138/28,405)	
	Male		40.71 (57,553/141,386)	40.97 (46,286/112,981)	39.67 (11,267/28,405)	
**Pregnancy status (among females <50 years of age), % (n/N)**						<.001
	Yes, currently pregnant, planning pregnancy, or breastfeeding		5.64 (2782/49,344)	5.92 (2192/37,003)	4.78 (590/12,341)	
	Not pregnant		94.36 (46,562/49,344)	94.08 (34,811/37,003)	95.22 (11,751/12,341)	
**Age (years)**						<.001^b^
	Mean (SD)		44.4 (13.8)	45.3 (14.0)	40.8 (12.8)	
	Range		18-98	18-98	18-93	
	**Age group, % (n/N)**					
		18-24	8.95 (12,663/141,427)	8.57 (9682/113,017)	10.49 (2981/28,410)	
		25-34	19.42 (27,465/141,427)	17.72 (20,028/113,017)	26.18 (7437/28,410)	
		35-44	19.34 (27,352/141,427)	18.22 (20,589/113,017)	23.80 (6763/28,410)	
		45-54	27.11 (38,336/141,427)	27.99 (31,630/113,017)	23.60 (6706/28,410)	
		55-64	18.19 (25,722/141,427)	19.60 (22,151/113,017)	12.57 (3571/28,410)	
		≥65	6.99 (9889/141,427)	7.91 (8937/113,017)	3.35 (952/28,410)	
**Race/ethnicity, % (n/N)**						<.001^b^
	White, non-Hispanic		72.90 (101,492/139,220)	71.10 (79,160/111,340)	80.10 (22,332/27,880)	
	Black or African American, non-Hispanic		13.20 (18,382/139,220)	14.80 (16,476/111,340)	6.84 (1906/27,880)	
	Hispanic or Latino		7.47 (10,405/139,220)	7.39 (8226/111,340)	7.82 (2179/27,880)	
	Other		6.42 (8941/139,220)	6.72 (7478/111,340)	5.25 (1463/27,880)	
**Education, % (n/N)**						<.001^b^
	< High school degree		17.90 (24,807/138,620)	19.96 (22,087/110,681)	9.74 (2720/27,939)	
	General education development (GED)		7.43 (10,298/138,620)	7.21 (7979/110,681)	8.30 (2319/27,939)	
	High school degree		27.40 (37,983/138,620)	28.69 (31,749/110,681)	22.31 (6234/27,939)	
	> High school		47.27 (65,532/138,620)	44.15 (48,866/110,681)	59.65 (16,666/27,939)	
**Health insurance status, % (n/N)**						<.001^b^
	Uninsured		42.62 (59,432/139,461)	41.00 (45,688/111,422)	49.02 (13,744/28,039)	
	Commercial		25.47 (35,525/139,461)	22.14 (24,668/111,422)	38.72 (10,857/28,039)	
	Medicaid		19.25 (26,853/139,461)	22.12 (24,647/111,422)	7.87 (2206/28,039)	
	Medicare		12.66 (17,651/139,461)	14.74 (16,419/111,422)	4.39 (1232/28,039)	
**Marital status,** ^c^ **% (n/N)**						<.001^b^
	Single		36.13 (17,867/49,455)	36.64 (15,823/43,183)	32.59 (2044/6272)	
	Married or domestic partner		36.04 (17,826/49,455)	34.63 (14,954/43,183)	45.79 (2872/6272)	
	Divorced, separated, or widowed		27.83 (13,762/49,455)	28.73 (12,406/43,183)	21.62 (1356/6272)	
**Annual household income (US$),** ^d^ **% (n/N)**						<.001^b^
	<$15,000		51.45 (21,073/40,961)	54.39 (18,614/34,224)	36.50 (2459/6737)	
	$15,000 to $35,000		31.30 (12,819/40,961)	30.11 (10,304/34,224)	37.33 (2515/6737)	
	>$35,000		17.26 (7069/40,961)	15.50 (5306/34,224)	26.17 (1763/6737)	
**Sexual orientation,** ^e^ **% (n/N)**						.13
	Heterosexual		93.86 (99,315/105,814)	93.92 (78,244/83,312)	93.64 (21,071/22,502)	
	Lesbian, gay, bisexual, transgender, or other		6.14 (6499/105,814)	6.08 (5068/83,312)	6.36 (1431/22,502)	
**Chronic health conditions, % (n/N)**						<.001^b^
	None		67.38 (94,919/140,862)	65.26 (73,611/112,798)	75.93 (21,308/28,064)	
	≥1^f^		32.62 (45,943/140,862)	34.74 (39,187/112,798)	24.07 (6756/28,064)	
**Mental health conditions,** ^g^ **% (n/N)**						<.001^b^
	None		53.41 (65,466/122,579)	50.58 (49,269/97,404)	64.34 (16,197/25,175)	
	≥1^h^		46.59 (57,113/122,579)	49.42 (48,135/97,404)	35.66 (8978/25,175)	
**Tobacco environment (other tobacco users present),** ^e^ **% (n/N)**						<.001^b^
	Home and/or work		56.99 (67,022/117,612)	56.24 (58,666/104,308)	62.81 (8356/13,304)	
	Neither home nor work		43.01 (50,590/117,612)	43.76 (45,642/104,308)	37.19 (4948/13,304)	
**Years used tobacco, % (n/N)**						<.001^b^
	<20 years		33.14 (44,268/133,589)	30.66 (32,590/106,284)	42.77 (11,678/27,305)	
	≥20 years		66.86 (89,321/133,589)	69.34 (73,694/106,284)	57.23 (15,627/27,305)	
**Dependence (time to first tobacco use after waking), % (n/N)**						<.001^b^
	Within 5 minutes		48.32 (65,575/135,716)	49.86 (53,927/108,154)	42.26 (11,648/27,562)	
	≥6 minutes		51.68 (70,141/135,716)	50.14 (54,227/108,154)	57.74 (15,914/27,562)	
**Tobacco type,** ^i^ **% (n/N)**						
	Cigarette		96.05 (135,593/141,174)	95.97 (108,237/112,783)	96.35 (27,356/28,391)	<.01
	Smokeless tobacco		4.02 (5679/141,174)	3.74 (4215/112,783)	5.16 (1464/28,391)	<.001
	Cigar		4.57 (6448/141,174)	4.55 (5136/112,783)	4.62 (1312/28,391)	.63
	Pipe		0.44 (628/141,174)	0.39 (445/112,783)	0.64 (183/28,391)	<.001
	Other		0.97 (1365/141,174)	0.79 (893/112,783)	1.66 (472/28,391)	<.001
**Cigarettes per day**						<.001
	Mean (SD)		19.0 (11.3)	19.1 (11.6)	18.4 (10.0)	
	Range		0-100	0-100	0-100	
**Cigarette frequency,** ^e^ **% (n/N)**						<.001
	Every day		94.02 (121,360/129,083)	93.68 (96,225/102,720)	95.34 (25,135/26,363)	
	Some days		2.36 (3044/129,083)	2.27 (2329/102,720)	2.71 (715/26,363)	
	Not at all		3.62 (4679/129,083)	4.06 (4166/102,720)	1.95 (513/26,363)	
**Method of entry into program, % (n/N)**						<.001^b^
	Fax referral		4.61 (6515/141,429)	5.17 (5842/113,019)	2.37 (673/28,410)	
	Phone call		82.59 (116,801/141,429)	91.93 (103,893/113,019)	45.43 (12,908/28,410)	
	Web enroll		12.81 (18,113/141,429)	2.91 (3284/113,019)	52.20 (14,829/28,410)	
**Treatment intensity, % (n/N)**						n/a
	Multiple-call		10.67 (15,087/141,429)	13.35 (15,087/113,019)	0.00 (0/28,410)	
	1-call		67.69 (95,728/141,429)	84.70 (95,728/113,019)	0.00 (0/28,410)	
	Switch from Web to multiple-call phone		1.56 (2204/141,429)	1.95 (2204/113,019)	0.00 (0/28,410)	
	Web-Only		20.09 (28,410/141,429)	0.00 (0/113,019)	100.00 (28,410/28,410)	
**Calls completed**			n/a^j^		n/a^j^	n/a^j^
	Mean (SD)			1.6 (1.1)		
	Range			0-10		
	**Number of calls completed, % (n/N)**					
		0 calls		10.13 (11,445/113,019)		
		1 call		53.87 (60,878/113,019)		
		2 calls		19.03 (21,512/113,019)		
		3 calls		9.41 (10,638/113,019)		
		4 calls		5.82 (6575/113,019)		
		≥5 calls		1.74 (1971/113,019)		

^a^ Responses of “refused,” “don’t know,” and “not collected” were excluded from analyses and resulted in different N’s for each analysis.

^b^ Met meaningful significance threshold requirements of *P*<.0001 and absolute difference in percentage points between comparison groups rounded to 5 or greater.

^c^ Marital status was assessed at enrollment by 3 states; analysis focused on a limited sample.

^d^ Annual household income was assessed at enrollment by 5 states; analysis focused on a limited sample.

^e^ Sexual orientation, tobacco environment, and cigarette frequency were assessed at enrollment by 9 states; analyses focused on a limited sample.

^f^ Endorsed 1 or more of the following chronic health conditions: asthma, diabetes, coronary artery disease, chronic obstructive pulmonary disease.

^g^ Six states (87.4% of study sample) assessed mental health condition status at enrollment by asking the question, “Do you currently have any mental health conditions, such as attention deficit hyperactivity disorder (ADHD), bipolar disorder, depression, drug or alcohol use disorder (substance use disorder; SUD), generalized anxiety disorder, posttraumatic stress disorder (PTSD), schizophrenia?” Analysis focused on a limited sample.

^h^ Endorsed 1 or more of the mental health conditions assessed.

^i^ Multiple reporting; total may not add up to 100%.

^j^ Coaching calls were not included in the Web-Only program.

### Characteristics of Web-Only Versus Phone/Web Enrollees

Quitline registrants were more likely to select Phone/Web over a Web-Only program (113,019/141,429, 79.91% vs 28,410/141,429, 20.09%). Participant characteristics and differences between those who opted for the Web-Only versus Phone/Web program are shown in Table 1.

Compared to Phone/Web enrollees, participants who enrolled in a Web-Only program were younger (mean 40.8, SD 12.8 vs mean 45.3, SD 14.0 years; *P*<.001), more likely to be white non-Hispanic (80.10% vs 71.10%; *P*<.001) and less likely to be black or African American non-Hispanic (6.84% vs 14.80%; *P*<.001). Web-Only enrollees were more highly educated (59.65% vs 44.15% had greater than a high school degree; *P<*.001) and reported higher household incomes (36.50% vs 54.39% reported an annual household income of less than US$15,000; 37.33% vs 30.11% reported US$15,000 to US$35,000; 26.17% vs 15.50% reported greater than US$35,000; all *P<*.001). Participants who opted for the Web-Only program were more likely to be uninsured (49.02% vs 41.00%; *P<*.001) or commercially insured (38.72% vs 22.14%; *P<*.001), and less likely to have Medicaid (7.87% vs 22.12%; *P<*.001) or Medicare coverage (4.39% vs 14.74%; *P<*.001). Web-Only were also more likely to be married or in a domestic partnership (45.79% vs 34.63%; *P<*.001), and more likely to live and/or work with other tobacco users (62.81% vs 56.24%; *P<*.001). Smaller proportions of Web-Only enrollees reported having a chronic health condition (24.07% vs 34.74%; *P<*.001) or a mental health condition (35.66% vs 49.42%; *P<*.001) at enrollment. Web-Only were also less likely to be highly nicotine-addicted (42.26% vs 49.86% reported using tobacco within 5 minutes of waking; *P<*.001) or long-term tobacco users (57.23% vs 69.34% had used tobacco for ≥20 years; *P<*.001). Web-Only were less likely to have started their enrollment for quitline services over the phone (45.43% vs 91.93%; *P<*.001) and more likely to have started their enrollment online (52.20% vs 2.91%; *P<*.001). There were no meaningful differences in program selection as a function of gender, pregnancy status, sexual orientation, cigarettes smoked per day, or frequency of cigarette use at enrollment.

As shown in Table 2, multivariable logistic regression analyses confirmed that participants who opted to enroll in Web-Only were younger, more highly educated, more likely to be white non-Hispanic and less likely to be black non-Hispanic, more likely to be uninsured or commercially insured, less likely to be highly nicotine dependent or have a chronic health condition, and more likely to have started their enrollment online.

**Table 2:** Multivariable model of the relationship of participant characteristics and program choice between Phone/Web versus Web-Only programs.

Baseline characteristic		Chose Web-Only program	
		AOR (99.99% CI)	*P*
Age		0.983 (0.981-0.986)	<.001^a^
**Gender**			
	Male	Ref	<.001^a^
	Female	1.106 (1.028-1.189)	
**Race/ethnicity**			
	Black or African American, non-Hispanic	Ref	
	White, non-Hispanic	1.681 (1.482-1.907)	<.001^a^
	Hispanic or Latino	1.308 (1.094-1.564)	
	Other	1.462 (1.216-1.758)	
**Education**			
	< High school degree	Ref	
	General education development (GED)	1.411 (1.202-1.656)	<.001^a^
	High school degree	1.481 (1.314-1.669)	
	> High school	1.792 (1.604-2.003)	
**Health insurance status**			
	Medicaid	Ref	
	Commercial	1.594 (1.415-1.794)	<.001^a^
	Uninsured	1.380 (1.237-1.541)	
	Medicare	0.945 (0.803-1.112)	
**Chronic health conditions**			
	≥1^b^	Ref	<.001^a^
	None	1.234 (1.137-1.340)	
Cigarettes per day		0.998 (0.995-1.002)	.06
**Dependence level**			
	Within 5 minutes	Ref	<.001^a^
	≥6 minutes	1.127 (1.046-1.213)	
**Method of entry**			
	Phone call	Ref	
	Web enroll	26.710 (24.270-29.396)	<.001^a^
	Fax referral	1.323 (1.102-1.589)	

^a^ Met meaningful significance threshold of *P*<.0001.

^b^ Endorsed 1 or more of the following chronic health conditions: asthma, diabetes, coronary artery disease, chronic obstructive pulmonary disease.

Multivariable analyses also indicated that Web-Only enrollees were more likely to be female, which was significant in bivariate analyses but did not meet our meaningful significance threshold requirement of an absolute difference in percentage points rounding to 5 or greater. A secondary model (not shown) limited to the 6 states that assessed mental health condition status at registration confirmed that Web-Only were also more likely to report not having any mental health condition diagnoses (AOR 1.49, 99.99% CI 1.38-1.61; *P*<.001).

### Utilization of Web Services and Nicotine Replacement Therapy Benefit

Table 3 summarizes Web utilization overall and between program types. Half (50.34%) of Phone/Web and all (100.00%) Web-Only registrants “enrolled” in Web services by providing their email address and consenting to be contacted via email. Among those who consented to this enrollment step, Web-Only registrants were significantly more likely than Phone/Web to log in to the Web Coach website (*users*) in the 6 months following their registration (76.85% vs 42.04%; *P<*.001). Although Web-Only were more likely to log in at least once, this group was less likely to return to the site on a later day (*return users*) compared to Phone/Web (40.15% vs 58.39%; *P<*.001). Among program participants who used the Web Coach website at least once, Phone/Web participants logged in on more days than Web-Only participants (median 2.0, IQR 1-4 vs median 1.0, IQR 1-2; *P<*.001).

**Table 3:** Web Coach website enrollment rates, log-in days, return rates, and receipt of nicotine replacement therapy (NRT) benefit among total sample and between Phone/Web versus Web-Only programs.

Utilization metric		Total N=141,429	Phone/Web n=113,019	Web-Only n=28,410	*P*
Enrolled in Web Coach website by providing email address and consenting to contact via email (among all participants), % (n/N)		60.31 (85,302/141,429)	50.34 (56,892/113,019)	100.00 (28,410/28,410)	<.001^a^
Logged in to Web Coach website (among enrolled), % (n/N)		53.64 (45,752/85,302)	42.04 (23,920/56,892)	76.85 (21,832/28,410)	<.001^a^
**Web Coach website log-in days (among enrolled), % (n/N)**					<.001^a^
	0 days	46.36 (39,550/85,302)	57.96 (32,972/56,892)	23.15 (6578/28,410)	
	1 day	26.99 (23,020/85,302)	17.50 (9954/56,892)	45.99 (13,066/28,410)	
	2 days	9.22 (7866/85,302)	7.42 (4220/56,892)	12.83 (3646/28,410)	
	3 days	4.55 (3877/85,302)	4.12 (2346/56,892)	5.39 (1531/28,410)	
	4 days	2.68 (2287/85,302)	2.56 (1455/56,892)	2.93 (832/28,410)	
	≥5 days	10.20 (8702/85,302)	10.45 (5945/56,892)	9.70 (2757/28,410)	
Returned to Web Coach website after initial log-in day (among logged in), % (n/N)		49.69 (22,732/45,752)	58.39 (13,966/23,920)	40.15 (8766/21,832)	<.001^a^
Web Coach website log-in days (among logged in), Median (IQR)		1.0 (1-3)	2.0 (1-4)	1.0 (1-2)	<.001^a^
**NRT benefit shipped,** ^b^ **% (n/N)**					<.001^a^
	Sent NRT	68.04 (96,229/141,429)	73.58 (83,159/113,019)	46.00 (13,070/28,410)	
	Not sent NRT	31.96 (45,200/141,429)	26.42 (29,860/113,019)	54.00 (15,340/28,410)	
**NRT benefit shipped** ^c^ **(among states offering NRT through Phone/Web and Web-Only), % (n/N)**					<.001^a^
	Sent NRT	75.56 (84,530/111,875)	83.11 (71,460/85,987)	50.49 (13,070/25,888)	
	Not sent NRT	24.44 (27,345/111,875)	16.89 (14,527/85,987)	49.51 (12,818/25,888)	

^a^ Met meaningful significance threshold requirements of *P*<.0001 and absolute difference in percentage points between comparison groups rounded to 5 or greater.

^b^ Analysis included total sample, regardless of whether or not states offered an NRT benefit through their Phone/Web and/or Web-Only programs.

^c^ Analysis focused on the 6 states that offered an NRT benefit through both their Phone/Web and Web-Only programs.

Six states offered an NRT benefit through both their Phone/Web and Web-Only programs. In these states, Web-Only enrollees were significantly less likely to have received NRT from their quitline (50.49% vs 83.11%; *P<*.001). Among all 10 states (regardless of whether NRT was offered through either program), 46.00% of Web-Only versus 73.58% of Phone/Web were sent quitline NRT.

### Return Users: Subpopulations More Likely to Return to Web Services

Table 4 shows the percentages of different subpopulations within Phone/Web and Web-Only logging in to the Web Coach website on 2 or more days (ie, *return users*). Among both Phone/Web and Web-Only, participants who were female, more highly educated, had used tobacco for 20 years or longer, were sent NRT through their quitline, or had Medicare coverage were more likely to return to the site after their initial log-in day (Table 4). Older participants were also more likely to return among both Phone/Web (age of return users: mean 43.0, SD 13.0 vs age of nonreturn users: mean 40.5, SD 12.8; *P<*.001) and Web-Only (mean 41.9, SD 12.7 vs mean 39.8, SD 12.5; *P<*.001). Phone/Web participants were also more likely to return if they had reported a higher household income or identified as white non-Hispanic or “other” race at registration; these differences in return users as a function of income and race/ethnicity were not observed in the Web-Only population. Trends in return users also differed between program groups as a function of smoking frequency reported at enrollment. Phone/Web enrollees who smoked cigarettes every day or some days at enrollment were more likely to return than those who reported smoking not at all. The opposite was found for the Web-Only population: enrollees who initially reported not smoking at all were more likely to return than Web-Only enrollees who smoked daily or only some days. For Phone/Web, participants who completed more coaching calls were also more likely to return to the Web Coach website. There were no meaningful differences in return rates that met our threshold for significance for either program group as a function of pregnancy status, marital status, chronic health or mental health condition status, tobacco environment, or nicotine dependence at enrollment.

Multivariable analyses confirmed that Phone/Web and Web-Only participants who were older, female, more highly educated, and received NRT from their quitline were more likely to return to the Web Coach website (Table 5). Patterns of return as a function of race/ethnicity were also confirmed: Phone/Web participants were more likely to return if they identified as white non-Hispanic or “other” race, whereas no difference was observed among Web-Only. Phone/Web were also significantly more likely to return if they were commercially insured and had completed at least 1 call with Quit Coach staff. Among Web-Only, differences in return rates between insurance groups did not meet our significance threshold. Web-Only were less likely to return if they started their enrollment online rather than over the phone; there were no differences in return rates by method of program entry for Phone/Web. As in bivariate analyses, there were no differences in return rates within either program as a function of chronic condition status or nicotine dependence.

**Table 4:** Subpopulations in the Phone/Web and Web-Only programs more likely to return to the Web Coach website after an initial log-in day.^a^

Baseline characteristic or program component		Phone/Web			Web-Only		
		%	(# Returned/subgroup n)	*P*	%	(# Returned/subgroup n)	*P*
Overall		58.39	(13,966/23,920)		40.15	(8766/21,832)	
**Gender**				<.001^b^			<.001^b^
	Female	60.26	(8760/14,537)		42.30	(5591/13,216)	
	Male	55.48	(5202/9376)		36.86	(3174/8612)	
**Pregnancy status (among females <50 years of age)**				<.001			.81
	Yes, currently pregnant, planning pregnancy, or breastfeeding	51.03	(273/535)		40.09	(170/424)	
	Not pregnant	58.39	(5391/9232)		40.68	(3755/9230)	
**Race/ethnicity**				<.001^b^			.26
	White, non-Hispanic	60.59	(10,369/17,114)		40.54	(7010/17,290)	
	Black or African American, non-Hispanic	51.86	(1435/2767)		39.33	(542/1378)	
	Hispanic or Latino	50.74	(1307/2576)		40.10	(719/1793)	
	Other	59.87	(719/1201)		37.61	(378/1005)	
**Education**				<.001^b^			<.001^b^
	< High school degree	52.78	(1217/2306)		31.94	(611/1913)	
	General education development (GED)	56.09	(810/1444)		33.08	(573/1732)	
	High school degree	56.14	(3188/5679)		37.98	(1767/4653)	
	> High school	60.61	(8566/14,132)		43.20	(5709/13,215)	
**Health insurance status**				<.001^b^			<.001^b^
	Uninsured	57.09	(5767/10,102)		37.74	(3999/10,597)	
	Commercial	60.24	(4377/7266)		42.52	(3630/8538)	
	Medicaid	54.85	(2165/3947)		38.36	(600/1564)	
	Medicare	64.86	(1460/2251)		48.54	(417/859)	
**Marital status** ^c^				.04			<.01
	Single	69.17	(821/1187)		32.08	(385/1200)	
	Married or domestic partner	72.45	(1270/1753)		36.92	(687/1861)	
	Divorced, separated, or widowed	73.76	(711/964)		32.48	(266/819)	
**Annual household income (US$)** ^d^				<.001^b^			.22
	<$15,000	60.03	(952/1586)		36.76	(569/1548)	
	$15,000 to $35,000	65.62	(1002/1527)		39.52	(677/1713)	
	>$35,000	68.40	(844/1234)		39.20	(490/1250)	
**Sexual orientation** ^e^				<.01			.41
	Heterosexual	56.96	(11,129/19,537)		40.89	(6885/16,836)	
	Lesbian, gay, bisexual, transexual, or other	61.15	(1012/1655)		42.12	(497/1180)	
**Chronic health conditions**				<.001			.09
	None	57.69	(9999/17,333)		39.91	(6596/16,526)	
	≥1^f^	60.41	(3942/6525)		41.25	(2078/5037)	
**Mental health conditions** ^g^				.08			.21
	None	57.57	(6340/11,013)		39.57	(5024/12,698)	
	≥1^h^	58.78	(5420/9221)		40.50	(2731/6744)	
**Tobacco environment (other tobacco users present)** ^e^				<.001			<.01
	Home and/or work	58.41	(7074/12,111)		40.25	(2425/6025)	
	Neither home nor work	60.86	(5240/8610)		43.54	(1558/3578)	
**Years used tobacco**				<.001^b^			<.001^b^
	<20 years	53.92	(4637/8600)		36.44	(3280/9002)	
	≥20 years	61.40	(8433/13,735)		42.41	(5063/11,938)	
**Dependence (time to first tobacco use after waking)**				<.001			<.001
	Within 5 minutes	57.57	(5972/10,373)		38.58	(3406/8828)	
	≥6 minutes	59.87	(7611/12,712)		40.86	(5053/12,367)	
**Cigarette frequency** ^e^				<.001^b^			<.001^b^
	Every day	58.95	(12,071/20,478)		39.69	(7662/19,307)	
	Some days	58.31	(256/439)		37.96	(205/540)	
	Not at all	45.36	(308/679)		50.14	(184/367)	
**Method of entry into program**				<.001^b^			<.001
	Fax referral	55.09	(379/688)		37.98	(109/287)	
	Phone call	59.45	(12,685/21,337)		41.80	(3989/9543)	
	Web enroll	47.60	(902/1895)		38.89	(4668/12,002)	
**Phone program intensity**				<.001^b^	n/a^i^	n/a^i^	n/a^i^
	1-call	48.34	(842/1742)				
	Multiple-call	59.18	(13,124/22,178)				
**Call completion**				<.001^b^	n/a^i^	n/a^i^	n/a^i^
	0 calls	16.39	(362/2208)				
	1 call	55.99	(6989/12,482)				
	2 calls	66.91	(3171/4739)				
	3 calls	74.51	(1815/2436)				
	4 calls	78.68	(1336/1698)				
	≥5 calls	82.07	(293/357)				
**NRT benefit shipped** ^j^				<.001^b^			<.001^b^
	Sent NRT	63.24	(11,988/18,957)		48.63	(6325/13,006)	
	Not sent NRT	39.85	(1978/4963)		27.66	(2441/8826)	
**NRT benefit shipped** ^k^ **(among states offering NRT through Phone/Web and Web-Only)**				<.001^b^			<.001^b^
	Sent NRT	62.20	(10,523/16,917)		48.63	(6325/13,006)	
	Not sent NRT	27.76	(855/3080)		26.16	(1907/7289)	

^a^ Analyses focused on those who logged in to the Web Coach website at least once. Responses of “refused,” “don’t know,” and “not collected” were excluded from analyses and resulted in different N’s for each analysis.

^b^ Met meaningful significance threshold requirements of *P*<.0001 and absolute difference in percentage points between comparison groups rounded to 5 or greater.

^c^ Marital status was assessed at enrollment by 3 states; analysis focused on a limited sample.

^d^ Annual household income was assessed at enrollment by 5 states; analysis focused on a limited sample.

^e^ Sexual orientation, tobacco environment, and cigarette frequency were assessed at enrollment by 9 states; analyses focused on a limited sample.

^f^ Endorsed ≥1 of the following chronic health conditions: asthma, diabetes, coronary artery disease, chronic obstructive pulmonary disease.

^g^ Six states (87.4% of study sample) assessed mental health condition status at registration by asking the question, “Do you currently have any mental health conditions, such as attention deficit hyperactivity disorder (ADHD), bipolar disorder, depression, drug or alcohol use disorder (substance use disorder; SUD), generalized anxiety disorder, PTSD, schizophrenia?” Analysis focused on a limited sample.

^h^ Endorsed ≥1 of the mental health conditions assessed.

^i^ Coaching calls were not included in the Web-Only program.

^j^ Analysis included total sample, regardless of whether or not states offered an NRT benefit through their Phone/Web and/or Web-Only programs.

^k^ Analysis focused on the 6 states that offered an NRT benefit through both their Phone/Web and Web-Only programs.

**Table 5:** Multivariable models of the relationship of participant characteristics and likelihood of returning to the Web Coach website within Phone/Web and Web-Only programs.^a^

Baseline characteristic or program component		Phone/Web return users		Web-Only return users	
		AOR (99.99% CI)	*P*	AOR (99.99% CI)	*P*
Age		1.011 (1.006-1.016)	<.001^b^	1.013 (1.008-1.018)	<.001^b^
**Gender**					
	Male	Ref	<.001^b^	Ref	<.001^b^
	Female	1.216 (1.078-1.373)		1.252 (1.105-1.419)	
**Race/ethnicity**					
	Black or African American, non-Hispanic	Ref		Ref	
	White, non-Hispanic	1.410 (1.176-1.690)	<.001^b^	0.947 (0.738-1.216)	.50
	Hispanic or Latino	1.151 (0.902-1.469)		1.001 (0.728-1.377)	
	Other	1.464 (1.071-2.002)		0.891 (0.616-1.289)	
**Education**					
	< High school degree	Ref		Ref	
	General education development (GED)	1.132 (0.847-1.513)	<.001^b^	1.055 (0.783-1.422)	<.001^b^
	High school degree	1.063 (0.857-1.318)		1.206 (0.945-1.539)	
	> High school	1.277 (1.046-1.557)		1.501 (1.202-1.874)	
**Health insurance status**					
	Medicaid	Ref		Ref	
	Commercial	1.245 (1.034-1.499)	<.001^b^	1.183 (0.914-1.531)	<.001^b^
	Uninsured	1.109 (0.936-1.314)		1.009 (0.785-1.296)	
	Medicare	1.167 (0.910-1.496)		1.192 (0.814-1.746)	
**Chronic health conditions**					
	≥1^c^	Ref	.50	Ref	.67
	None	1.024 (0.896-1.170)		0.985 (0.854-1.136)	
Cigarettes per day		0.999 (0.993-1.005)	.36	0.995 (0.988-1.001)	.002
**Dependence level**					
	Within 5 minutes	Ref	.001	Ref	.33
	≥6 minutes	1.106 (0.979-1.249)		1.033 (0.909-1.173)	
**Method of entry into program**					
	Phone call	Ref		Ref	
	Web enroll	0.919 (0.734-1.151)	.33	0.804 (0.702-0.920)	<.001^b^
	Fax referral	0.959 (0.664-1.386)		0.862 (0.496-1.499)	
**NRT benefit shipped** ^d^					
	Not sent NRT	Ref	<.001^b^	Ref	<.001^b^
	Sent NRT	1.828 (1.490-2.242)		3.091 (2.692-3.550)	
**Phone program intensity**					
	1-call	Ref	<.001	n/a^e^	n/a^e^
	Multiple-call	1.375 (1.000-1.893)			
**Call completion**					
	0 calls	Ref	<.001^b^	n/a^e^	n/a^e^
	≥1 call	4.599 (3.360-6.296)			

^a^ Analyses focused on those who logged in to the Web Coach website at least once.

^b^ Met meaningful significance threshold of *P*<.0001.

^c^ Endorsed ≥1 of the following chronic health conditions: asthma, diabetes, coronary artery disease, chronic obstructive pulmonary disease.

^d^ Analysis included total sample, regardless of whether or not states offered an NRT benefit through their Phone/Web and/or Web-Only programs.

^e^ Coaching calls were not included in the Web-Only program.

### Web Coach Website Feature Use

**Figure 8:**
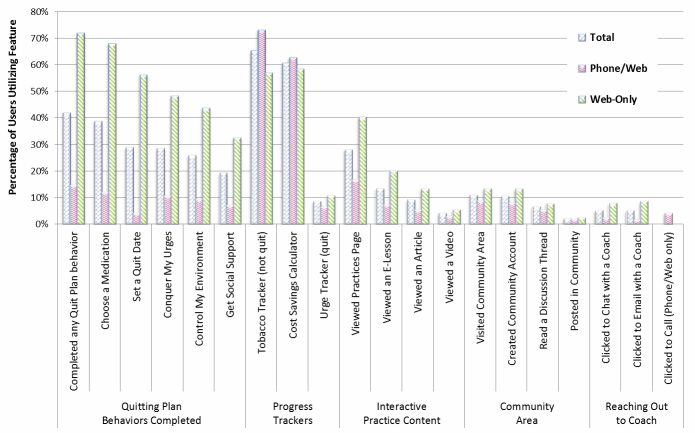
Participants using key Web Coach website features among total users and between Phone/Web versus Web-Only users.

Figure 8 and Table 6 show the percentages of Web Coach website users (logged in on 1 or more days) who used key site features at least once in the 6 months following their enrollment in a program. Table 6 shows all percentages and *P* values, whereas Figure 8 is included to facilitate synthesis of findings. Features used by the largest percentages of participants overall included the Tobacco Tracker (65.33% of all Web Coach website users) and the Cost Savings Calculator (60.64%). Participants also completed Quitting Plan behaviors at relatively high rates; 41.91% of all users completed at least 1 behavior: Choose a Medication (38.68%), Set a Quit Date (28.93%), Conquer My Urges (28.54%), Control My Environment (25.75%), and Get Social Support (19.22%). Although 27.92% of users visited the page introducing the 4 Essential Practices of Quitting, less than half of those individuals viewed any of the Practices content in an e-lesson (13.31%), article (9.13%), or video (4.03%). Use of the Community features was also low, with 10.87% of all Web Coach website users visiting the Community area, 10.44% creating a Community account, 6.48% reading a discussion thread, and 1.99% posting in a Community discussion. Small proportions of users reached out to Quit Coach staff: 5.02% of all Phone/Web and Web-Only participants clicked to chat with a coach, and 4.85% clicked to send an email. Phone/Web participants could also request a call from a coach; 4.04% of Phone/Web used this feature.

With regard to differences in feature use between Phone/Web and Web-Only, Web-Only participants were less likely than Phone/Web to use both the Tobacco Tracker (56.87% vs 73.06%; *P<*.001) and Cost Savings Calculator (58.37% vs 62.72%; *P<*.001; only approached threshold for meaningful significance), but they were more likely to use all other key features, including completing the 5 Quitting Plan behaviors: Choose a Medication (68.05% vs 11.87%; *P<*.001), Set a Quit Date (56.17% vs 4.06%; *P<*.001), Conquer My Urges (48.22% vs 10.58%; *P<*.001), Control My Environment (43.75% vs 9.33%; *P<*.001), and Get Social Support (32.47% vs 7.12%; *P<*.001) (Figure 8 and Table 6). Web-Only participants were also slightly more likely to use the Urge Tracker (10.72% vs 6.42%; *P<*.001; only approached threshold for meaningful significance), which was available only when a participant had a self-reported status of “I’m Quit.”

**Table 6:** Participants using key Web Coach website features among total users and between Phone/Web versus Web-Only users.^a^

Website feature		Total users, n (%) (N=45,752 ^b^)	Phone/Web users, n (%) (n=23,920 ^b^)	Web-Only users, n (%) (n=21,832 ^b^)	*P*
**Quitting plan behaviors**					
	Completed any Quit Plan behavior	19,176 (41.91)	3475 (14.53)	15,701 (71.92)	<.001^c^
	Choose a Medication^d^	17,696 (38.68)	2840 (11.87)	14,856 (68.05)	<.001^c^
	Set a Quit Date	13,236 (28.93)	972 (4.06)	12,264 (56.17)	<.001^c^
	Conquer My Urges	13,058 (28.54)	2531 (10.58)	10,527 (48.22)	<.001^c^
	Control My Environment	11,782 (25.75)	2231 (9.33)	9551 (43.75)	<.001^c^
	Get Social Support	8793 (19.22)	1704 (7.12)	7089 (32.47)	<.001^c^
**Progress trackers**					
	Tobacco Tracker (not quit)	29,892 (65.33)	17,477 (73.06)	12,415 (56.87)	<.001^c^
	Cost Savings Calculator	27,745 (60.64)	15,002 (62.72)	12,743 (58.37)	<.001
	Urge Tracker (quit)	3877 (8.47)	1536 (6.42)	2341 (10.72)	<.001
**Interactive practice content**					
	Viewed Practices page	12,775 (27.92)	3984 (16.66)	8791 (40.27)	<.001^c^
	Viewed an e-lesson	6090 (13.31)	1715 (7.17)	4375 (20.04)	<.001^c^
	Viewed an article	4176 (9.13)	1266 (5.29)	2910 (13.33)	<.001^c^
	Viewed a video	1845 (4.03)	691 (2.89)	1154 (5.29)	<.001
**Community**					
	Visited Community area	4975 (10.87)	2074 (8.67)	2901 (13.29)	<.001^c^
	Created Community account	4776 (10.44)	1904 (7.96)	2872 (13.16)	<.001^c^
	Read a discussion thread	2966 (6.48)	1277 (5.34)	1689 (7.74)	<.001
	Posted in Community	912 (1.99)	407 (1.70)	505 (2.31)	<.001
**Reaching out to Quit Coach staff**					
	Clicked to chat with a Coach	2297 (5.02)	586 (2.45)	1711 (7.84)	<.001^c^
	Clicked to email with a Coach	2220 (4.85)	375 (1.57)	1845 (8.45)	<.001^c^
	Clicked to call a Coach (Phone/Web only)	n/a^e^	967 (4.04)	n/a^e^	n/a^e^

^a^ Analyses limited to those who logged in to the Web Coach website at least once.

^b^ Denominator applies to entire column.

^c^ Met meaningful significance threshold requirements of *P*<.0001 and absolute difference in percentage points between comparison groups rounded to 5 or greater.

^d^ The Choose a Medication behavior was available to all Web Coach website users to guide their medication selection and dosing, regardless of whether the participant’s state quitline offered cessation medication. In addition, Web-Only participants who completed the activity but had a medical use exclusion contraindicating NRT use were not sent NRT; Phone/Web participants with a use exclusion could receive NRT from the quitline with physician approval.

^e^ Coaching calls were not included in the Web-Only program.

## Discussion

### Principal Results and Comparison With Prior Work

This examination of tobacco users’ enrollment in and use of Phone/Web and Web-Only tobacco cessation programs through 1 of 10 state quitlines describes program selection in a large, real-world sample, and is also the first examination of return users from Web-based versus integrated Phone/Web programs and among subpopulations within these programs. Four-fifths of the 141,429 tobacco users in the study selected cessation support through the integrated Phone/Web program. Method of entry was the strongest predictor of program selection (92% of those who started their enrollment by phone selected Phone/Web; 52% of those who started enrollment online selected Web-Only), which may indicate that the phone program is highlighted more during phone registration, in quitline advertisements, and/or through word of mouth. It is also possible that participants tend to contact the program using the modality in which they would prefer to receive services.

Those who chose to enroll in the Web-Only program were younger, healthier (in terms of chronic health condition and mental health condition status), more highly educated, less likely to have insurance through Medicaid or Medicare, more likely to be white non-Hispanic and less likely to be black non-Hispanic, and less likely to be highly nicotine-addicted. This profile of smokers may be more tech savvy and not as interested in the more intensive support available via phone. These findings and previous research indicate that Web-Only is an attractive option for the harder-to-reach population of younger smokers [7,9] and those without symptoms of depression [9]. In addition to differences in education and insurance status, among 4 states that assessed income, Web-Only enrollees had higher annual household incomes, which may relate to socioeconomic disparities in Internet access or experience using computers. If Web-based services prove to be effective for tobacco users of higher socioeconomic status, this may present an avenue for conserving tobacco control funds to provide the higher-cost phone programs [7,12] for smokers who are more highly addicted and socioeconomically disadvantaged.

Phone/Web and Web-Only enrollees engaged with Web services differently. After initial enrollment in the Web Coach website, more Web-Only participants actually logged in to the program, as would be expected by their program selection. However, Phone/Web participants who used the site were more likely to return after their initial log-in. Despite being less likely to return to the site, Web-Only participants were more likely than Phone/Web to use most key features, most notably completing the 5 Quitting Plan behaviors. These findings suggest that Web-Only participants tended to use the site for a single, intense session of quit plan development and site exploration, but typically did not return. Phone/Web participants, on the other hand, who already demonstrated their desire for support and program contact by choosing a program with phone interaction, used the Web Coach website planning features at much lower rates (likely because they had already completed planning with Quit Coach staff over the phone), but were more likely to return to the site for additional program contact. Phone/Web participants may have returned to the site because they were encouraged to do so during their ongoing coaching calls (call completion was correlated with return visits to the site), although participants may have completed more calls and returned to the site simply because they were more engaged overall. These motivations and usage profiles should be considered as program designers decide how to present and highlight Web-based cessation content to ensure participants find the most important and relevant content during their visit.

Within both the Phone/Web and Web-Only populations, participants were more likely to return to the Web Coach website after their initial log-in day if they were women, older, or more highly educated, which is in-line with the previous research [13]. Those who were sent NRT through their quitline (for both programs) and those who had commercial health insurance (among the Phone/Web program) were also more likely to return. Given findings that services are most successful [14,15] and cost-effective when utilized at higher rates [12], effective strategies for re-engaging participants across the board, or particularly for less engaged groups, are needed.

The interactive Tobacco Tracker, Cost Savings Calculator, and Quitting Plan behaviors were the most widely used features among all registrants; previous research has suggested that use of interactive Web components is associated with higher quit rates, particularly in nondepressed populations [9,14]. Program designers should continue to focus on interactive features as opposed to static informational sites. Designers should also consider how best to encourage use of key features. Among quitlines that offered NRT to both Phone/Web and Web-Only participants, Web-Only were less likely to have received NRT from their quitline program. It is unclear why Web-Only participants were less likely to take advantage of the NRT benefit through their quitline, but it may be the result of different program processes. Although a Phone/Web participant is typically guided through the process of creating a quit plan (including selection and dosing for a cessation medication) by Quit Coach staff, the Web-Only program is designed to be more self-guided; participants are required to authenticate their account, log in, and then complete the Choose a Medication behavior on their own to access NRT. In addition, Phone/Web participants with a medical use exclusion contraindicating NRT use are mailed an override letter that their physician can fax to the quitline to approve NRT for the participant; this override process has not been an option for Web-Only participants with a use exclusion. Because use of Food and Drug Administration-approved cessation medications is associated with greater odds of achieving abstinence [16], Web programs should employ strategies to promote awareness of medication options and prioritize access to cessation medication benefits.

### Limitations and Future Directions

A strength of this study is that analyses were conducted with a large census sample of tobacco users from different regions of the country who registered in 10 state tobacco quitlines. However, the large sample size resulted in numerous statistically significant results that may not reflect meaningful differences; the authors used a Bonferroni adjustment to account for the large number of statistical comparisons and selected a criterion level to provide a consistent benchmark for identifying meaningful differences. We believe a 5 percentage point difference is a reasonable threshold; however, others may view smaller or larger differences to be meaningful.

Several other limitations should be noted. First, all participants self-selected their program of choice, but we do not know what factors influenced participants’ selections nor how aware tobacco users were of the services available to them in each program. In particular, we cannot know how carefully those who enrolled online read the program option descriptions. Second, not all 10 states offered identical services to all tobacco users (eg, the multiple-call program was not available to subgroups of registrants in 2 states); future work should examine the impact of different service offerings on program choice. Third, data were not available from Web-Only participants on motivation, confidence, readiness to quit, or previous quit attempts. These data would better inform whether individuals less ready or who might feel they needed less support with quitting selected the Web-Only service. It is important to note that the demographic differences found between participants who chose Web-Only versus Phone/Web may be due in part or entirely to differences in 1 or more of these variables for which data were not available. Fourth, we focused on the number of distinct log-in days as our metric of engagement and were not able to examine the specific number of log-ins or minutes on the site. Log-in days provides a more consistent estimate of use given potential variations in time before automatic logouts; however, log-ins and minutes could have provided additional context regarding typical program use. Fifth, we did not examine utilization of other resources; Web-Only users or other subgroups less likely to return to the Web Coach website may also be more likely to use other additional sources of support (eg, multiple online programs). Future research could examine this hypothesis and determine whether encouraging sustained engagement in a single evidence-based program produces the best outcomes. Sixth, there was not a mobile accessible/compatible version of the Web Coach website at the time of this study. Future research should examine whether mobile accessible websites change how participants engage with Web-based programs, especially in terms of differences in use between various levels of socioeconomic status. Moreover, because Web features can change, feature use could be impacted by changes in site design, which can affect the replicability of these findings and comparisons across Web-based programs.

This paper was not about outcomes; although the effectiveness of phone-based cessation programs has been established [17] and randomized controlled trials have provided support for Web-based cessation services [10], data were not available to evaluate the effectiveness of the Phone/Web and Web-Only programs for participants in this study, and the Centers for Disease Control and Prevention has not yet deemed Web-based services as having a sufficient evidence base in their 2014 Best Practice Guidelines [18]. More research is needed to better understand the effectiveness of Web-Only cessation services for populations who select into that service, and reasons tobacco users select Web-Only over integrated Phone/Web.

### Conclusions

Understanding who is selecting different tobacco cessation program modalities and how they engage with the program in a real-world setting will help the scientific and treatment community to better understand program outcomes and can inform engagement and re-engagement strategies. Our findings suggest that a Web-based program attracts younger, healthier smokers of higher socioeconomic status who interact more intensely with services in a single session, but are also less likely to re-engage or access the important NRT benefits available to them. Further research is needed to examine the efficacy of different engagement techniques and services with different subpopulations of tobacco users.

## Abbreviations

GED: general education development
NAQC: North American Quitline Consortium
NRT: nicotine replacement therapy
